# MOBILE HEALTH AND RESOURCE FAIRS TO ADDRESS SOCIAL DETERMINANTS OF HEALTH OF LOW-INCOME OLDER ADULTS

**DOI:** 10.1093/geroni/igae098.4242

**Published:** 2024-12-31

**Authors:** Angie Sardina, Jeffrey Meadows, Lena Simon, Marsha Hampton, Christine Phillips, Lesley Ross, Alyssa Gamaldo

**Affiliations:** Clemson University, Clemson, South Carolina, United States; Clemson University, Clemson, South Carolina, United States; Clemson University, Clemson, South Carolina, United States; Clemson University, Clemson, South Carolina, United States; Clemson University, Clemson, South Carolina, United States; Clemson University, Clemson, South Carolina, United States; Clemson University, Clemson, South Carolina, United States

## Abstract

Mobile Health and Resource Fairs (MHRFs) were designed to provide resources, education, and social engagement to low-income communities through reducing traditional barriers and constraints for older adults. We (1) explored effective marketing approaches and rationale for resident attendance; (2) examined satisfaction and expectations of the MHRFs, and (3) examined the likelihood for attending future events. MHRF events comprised 2 sites in Greenville, SC. Ninety-four participants (ages 50-90+) registered (site 1=77%; site 2=23%). Participants were asked to complete survey items on how they learned of the event and their reason for attending (aim 1). At the end of the MHRF, participants reported on event expectations and satisfaction (aim 2) using a Likert scale ranging from 0 (not all) to 10 (very much), and the likelihood of attending future events (aim 3) using a single Likert-Scale question ranging from 0 (very unlikely) to 4 (very likely). Attendees were predominately female (64%), Black/African American (73%), with a high school education (55%). Effective marketing approaches (aim 1) were event flyers (62%), other (14%; e.g., MHRF-specific staff/activities or housing activities/advertisements), walking by the event (11%), family/friends recommendations (9%), and housing staff recommendations (5.3%). Attendance rationale included obtaining healthy-aging information (49%), social engagement (28%), access to necessities (19.1%); and volunteerism (16%). Participants reported high expectations (M=9.72) and satisfaction (M=9.58) with the MHRF. Participants reported they were “somewhat-very likely” to attend future events (83%, aim 3). These findings affirm MHRFs can successfully provide critical health/social service resources, while simultaneously engaging older adults from underserved lower-income communities.

